# Point of view of the Italians pediatric scientific societies about the pediatric care during the COVID-19 lockdown: what has changed and future prospects for restarting

**DOI:** 10.1186/s13052-020-00907-3

**Published:** 2020-10-02

**Authors:** Riccardo Lubrano, Alberto Villani, Stefano Berrettini, Paolo Caione, Alberto Chiara, Antonella Costantino, Roberto Formigari, Emilio Franzoni, Guido Castelli Gattinara, Arturo Giustardi, Giancarlo La Marca, Paolo Lionetti, Mario Lima, Claudio Maffei, Monica Malamisura, Giantonio Manzoni, Gian Luigi Marseglia, Antonio Memeo, Fabio Mosca, Giovanna Perricone, Licia Peruzzi, Giorgio Piacentini, Gabriella Pozzobon, Enrica Riva, Simonetta Tesoro, Giuseppe Zampino, Federica Zanetto, Marco Zecca, Silvia Bloise

**Affiliations:** 1 Federazione Italiana delle Associazioni e Società Scientifiche dell’Area Pediatrica e Società Italiana di Emergenza Urgenza Pediatrica, Rome, Italy; 2 Dipartimento Materno Infantile e di Scienze Urologiche, Sapienza Università di Roma, UOC di Pediatria e Neonatologia, Ospedale Santa Maria Goretti, Polo di Latina, Latina, Italy; 3 Società Italiana di Pediatria, Rome, Italy; 4 Società Italiana di Otorinolaringologia Pediatrica, Rome, Italy; 5 Società Italiana di Videochirurgia Infantile, Rome, Italy; 6 Società Italiana di Pediatria Ospedaliera, Milan, Italy; 7 Società Italiana di Neuropsichiatria dell’Infanzia e dell’Adolescenza, Milan, Italy; 8 Società Italiana di Cardiologia Pediatrica e delle Cardiopatie Congenite, Firenze, Italy; 9 Società Italiana di Neurologia Pediatrica, Milan, Italy; 10 Società Italiana di Infettivologia Pediatrica, Milan, Italy; 11 Società Italiana per la Care in Perinatologia, Milan, Italy; 12 Società Italiana per lo Studio delle Malattie Metaboliche Ereditarie e lo Screening neonatale, Milan, Italy; 13 Società Italiana di Gastroenterologia Epatologia e Nutrizione Pediatrica, Milan, Italy; 14 Società Italiana di Chirurgia Pediatriaca, Milan, Italy; 15 Società Italiana di Endocrinologia e Diabetologia Pediatrica, Ferrara, Italy; 16 Osservatorio Nazionale Specializzandi in Pediatria, Rome, Italy; 17 Società Italiana di Urologia Pediatrica, Rome, Italy; 18 Società Italiana di Allergologia e Immunologia Pediatrica, Milan, Italy; 19 Società Italiana di Ortopedia e Traumatologia Pediatrica, Rome, Italy; 20 Società Italiana di Neonatologia, Rome, Italy; 21 Società Italiana di Psicologia Pediatrica, Rome, Italy; 22 Società Italiana di Nefrologia Pediatrica, Milan, Italy; 23 Società Italiana per le Malattie Respiratorie Infantili, Rome, Italy; 24 Società Italiana di Medicina dell’Adolescenza, Rome, Italy; 25 Società Italiana di Nutrizione Pediatrica, Milan, Italy; 26 Società di Anestesia e Rianimazione Neonatale e Pediatrica Italiana, Rome, Italy; 27 Società Italiana di Malattie Genetiche Pediatriche e Disabilità, Rome, Italy; 28 Associazione Culturale Pediatri, Narbolia, Italy; 29 Associazione Italiana di Ematologia e Oncologia Pediatrica, Rome, Italy

**Keywords:** COVID-19, Pediatric assistance, Children, Telemedicine

## Abstract

**Background:**

The coronavirus disease 2019 (COVID-19) is currently rare in children and they seem to have a milder disease course and better prognosis than adults. However, SARS-Cov-2 pandemic has indirectly caused problems in pediatric medical assistance. In view of this we wanted to draw a picture of what happened during health emergency and analyze future prospects for restarting.

**Methods:**

We involved the Italian pediatric scientific societies institutionally collected in the Italian Federation of Associations and Scientific Societies of the Pediatric Area (FIARPED); We sent a questionnaire to all scientific societies about the pediatric care activity during the COVID-19 emergency and future perspectives for the phase of post-containment.

**Results:**

The analysis of the questionnaires showed significant decrease of:admission, outpatient visits and specialist consultancy activities during the COVID-19 emergency, primarily linked to the fear of infection. Instead it was increased the serious degree of diseases admitted. Most of scientific societies maintained the relationship with chronic patients through some form of telemedicine, reporting a strong positive opinion about this modality. Finally showed the need to give life a new approach for hospitalizations and outpatient visits through a greater use of telemedicine, educational programs on families and a more decisive role of family pediatricians.

**Conclusions:**

Our study highlighted many aspects that can be improved in pediatric care. We think that It will be necessary a new shared strategy to improve the management and continuity of care for pediatric patients, primarily developing a network of collaboration between families, family pediatrician and hospitals and by enhancing the use of new methods of telecommunications**.**

## Introduction

The disease known as COVID-19, caused by the new coronavirus SARS-Cov-2, has recently been declared a pandemic by WHO [1], with a more favorable clinical course on children compared to the adults [2–4]. However, the SARS-CoV-2 pandemic has indirectly caused problems in the care of young patients. The COVID-19 outbreak, and the related attention from the media, has brought about intense psychological pressure on families. This, together with the Italian government’s lockdown orders, in place from March 9 to May 3, characterized by the suspension of common commercial activities and catering services, the closure of schools, the prohibition of groups of people in public places, and the banning of travel to municipalities other than those to which people belong, has caused a completely new and unexpected scenario in pediatric care. Consequently, several questions related to child’s health care have arisen.

In this context, we wanted to draw a picture of what happened among Italian pediatric assistance services and to examine operational proposals to plan a joint action for the post-COVID phase. To this end, we collected, through a questionnaire, the views of 28 Italian medical and surgical pediatric scientific societies belonging to the FIARPED (Italian Federation of Associations and Scientific Societies of the Pediatric Area).

The questionnaire was sent out in the first week of May, at the end of the lock down phase and before the start of the so-called “Phase 2”, characterized by a gradual loosening of the previous containment measures, since with the epidemic curve is being in a downward phase.

## Methods

The study has involved all 28 of the Italian pediatric scientific societies (Tables 1 and 2), 23 from the medical field and 5 from the surgical field, institutionally collected in the FIARPED. Each society was asked to fill in a questionnaire (Additional file 1, see below) about the pediatric care activity during the COVID-19 emergency and future perspectives for the phase of post-containment.

**Table 1:** Medical pediatric scientific societies collected in the FIARPED

Medical Area (23)	
Italian Society of Pediatrics -SIP	
Italian Society of Pediatric Psychology - SIPPed	
Italian Society of Pediatric Nephrology - SINePe	
Italian Society of Pediatric Infectivology - SITIP	
Italian Society for Perinatology Care - AICIP	
Cultural Association of Pediatricians - ACP	
Italian Association of Pediatric Hematology and Oncology -AIEOP	
National observatory of Pediatric residents- ONSP	
Italian Society of Pediatric Cardiology and Congenital Heart Disorders - SICP	
Italian Society of Pediatric Endocrinology and Diabetology - SIEDP	
Italian Society of Adolescent Medicine - SIMA	
Italian Society of Neonatology - SIN	
Italian Society of Pediatric Emergency and Urgent Medicine - SIMEUP	
Italian Society of Pediatric Genetic Diseases and Disabilities - SIMGePeD	
Italian Society of Hospital Pediatrics - SIPO	
Italian Society of Pediatric Neurology - SINP	
Italian Society of Gastroenterology Hepatology and Pediatric Nutrition - SIGENP	
Italian Society of Child and Adolescent Neuropsychiatry - SINPIA	
Italian Society for Infant Respiratory Diseases - SIMRI	
Italian Society of Pediatric Nutrition - SINUPE	
Italian Neonatal and Pediatric Anesthesia and Resuscitation Society - SARNePI	
Italian Society of Allergy and Pediatric Immunology-SIAIP	
Italian Society for the study of Hereditary Metabolic Diseases and Neonatal Screening-SIMMSEN

**Table 2:** Surgical pediatric scientific societies collected in the FIARPED

Surgical Area (5)	
Italian Society of Pediatric Urology - SIUP	
Italian Society of Orthopedics and Pediatric Traumatology - SITOP	
Italian Society of Infant Video Surgery - SIVI	
Italian Society of Pediatric Otorhinolaryngology - SIOP	
Italian Society of Pediatric Surgery - SICP	

The main issues of the questionnaire were: the percentage of reduction of hospitalizations, outpatient visits, new diagnoses and consultancy activities and continuity of care of chronic patients.

The reduction percentages were based on the opinion of the Presidents and of scientific Committees of the societies, in order to offer a global point of view on changes in specific areas of pediatric care during lock down.

In the second part of the questionnaire we analyzed the application of telemedicine in the various specialties and its possible advantages and the use of other alternative assistance methods and possible future assistance options.

### Statistical analysis

The statistical analysis was performed with JMP® 14.3.0 program for Mac (SAS Institute Inc). All data are expressed as median, 75° and 25° quantiles. Fisher’s test was used to evaluate the difference between the collected data and a *p* value < 0.05 was considered significant.

## Results

The results of the study are reported in Table 3. The results are expressed as median (25–75 quantiles).

**Table 3:** Main results of the questionnaire

Items	All societies	Medical area	Surgical area
**Reduction admissions (%)**	**70 (80–50)**	**60 (80–45)**	**80 (90–75)**
**Reduction outpatient visits (%)**	**80 (90–70)**	**80 (90–70)**	**80 (100–75)**
**Severity level of disease (1 to 5)**	**5 (5–4)**	**4 (5–4)**	**5 (5–4)**
**Reduction new diagnosis (%)**	**60 (75–25)**	**50 (70–30)**	**70 (85–70)**
**Admissions inappropriate pre-Covid (%)**	**20 (40–10)**	**30 (40–10)**	**10 (15–10)**
**Outpatient visit inappropriate pre- Covid (%)**	**20 (45–10)**	**30 (50–10)**	**20 (25–10)**
**Reduction of consultancy activities (%)**	**50 (72,5-37,5)**	**50 (70–30)**	**70 (80–45)**
**Reduction of own consultancy activities (%)**	**50 (72,5-37,5)**	**50 (70–30)**	**60 (80–45)**

All data are expressed as median and (75°-25°quantiles)

During the lock-down, all pediatric specialties had showed a reduction of admissions of about 70%; While, the reduction of outpatientvisits was of about 80%. These reductions are similar for medical area and surgical area. However, the severity of diseases admitted was more serious than in the pre-covid era, especially for surgical area. This phenomenon caused also the reduction of new diagnoses number of about 60%, with a higher percentage for the surgical area.

Only the activities of neonatology and pediatric oncology have not been affected by this phenomenon because they are linked to births and to the treatment protocols for cancer disease.

Therefore we also evaluated the main determinants in the reduction of the admissions: all medical and surgical societies of pediatrics have detected primarily parents’ fear to expose their children to a covid-19 infection by attending healthcare facilities (68%), secondary the revaluation by families about the clinical conditions which determine the need for hospitalization (32%) and finally the failure to be send by the family pediatrician to the hospital admission (21%). The main determinants in the reduction of outpatient visits were very similar: primarily the fear of infection (75%), secondary the revaluation by families about the clinical conditions which determine the need for pediatric visit (28%) and finally the difficulty to be visited by family pediatrician (25%). Furthermore in pre-covid era the median of hospitalizations and outpatient visits found to be inappropriate by pediatricians was of about 20%, with an higher percentage for the medical area.

The decrease of hospitalized patients has also determined a reduction of specialist consultancy activities of about 50%, without differences between the medical and surgical areas.

In this period of lack of communication with families, the 86% of scientific societies has reported that medical doctors have maintained the relationship between their clinical departments and their patients through some form of telemedicine, even if everyone has considered this method to be inadequate to replace the physical examination.

We also questioned ourselves about the role of telemedicine could have in home care: 57% of pediatric scientific societies answered that the telemedicine can be useful for patients with chronic conditions. The main telemedicine roles described was: primarily verbal consultation, secondary monitoring of one or more instrumental and biochemical parameters and only in small part to be an active part of an physical examination. The main ways of continue the care of chronic patients described “as telemedicine” from the pediatric scientific societies, were: by phone (68%) and video call (43%).

Subsequently we investigated what the prospects of welfare reorganization at the end of the containment for the normal practices for hospitalizations: 71% of scientific societies think that will be necessary to formulate a new approach methodology by specialist services, while 39% of scientific societies think that the family pediatrician will have to play a more decisive role regarding hospitalization needs and 20% of scientific societies think that everything will be like before. Finally all scientific societies have pointed out the need for a health education program for the family to guarantee a more appropriate and aware use of structures and services.

## Discussion

Our work showed a significant reduction in hospitalizations and outpatient visits in almost all pediatrics areas during this health emergency. The percentage of reduction of hospitalizations and outpatient visits was greater for the medical area than for surgery. The main determinants for this result were related to the fear of infection and to the revaluation by families about the clinical conditions of the child requiring medical evaluation. Results analysis has also shown a certain degree of inappropriateness of admissions and specialist visits in the pre-COVID-19 era. The phenomenon of inappropriate visits, especially in the emergency, for non-urgent problems is unfortunately widespread among many countries worldwide [5–7]. The reasons of this can be mostly in an inadequate hospital and territorial organization, the development of defensive medicine with excessive medical caution in the management of the patients and a reduced efficiency of primary care [8–14]. It’s clear that this excess of visits, in pre-covid era, could depend on a poor management capacity of parents, which required specialist visits for mild clinical conditions by creating overcrowding in pediatric emergency departments.

However, on the other side, even if the emergency room visits decreased considerably in this period, most children evaluated had severe illnesses and the percentage of patients hospitalized for serious diseases increased. Indeed, the analysis of the questionnaires revealed a lower reduction in consultancy (50%) compared to a reduction in hospitalizations (70%). The reason for this data could be a higher level of severity of patients hospitalized with the need for specialist consultations. The increase in severe admission could be explained also by the fear of coronavirus infection that caused a delay to medical consultation with consequent evaluation of patients already in critical condition.

In view of the difficulty in performing outpatient visits, during the COVID-19 emergency, the majority of pediatricians have maintained relationships with chronic patients through telephone consultation and videoconferencing. In fact, the analysis of questionnaires revealed a strong positive opinion about telemedicine, especially for chronic patients. Nonetheless, all pediatric societies agree that telemedicine cannot replace in-person visits in particular for an acute patient. In fact, there are little evidence about clinical outcomes and the effectiveness of telemedicine services for children [15].

However, the advantages by “telemedicine” reported by various societies are in agreement with those already described in the literature [15–18]. These advantages were represented primarily by a reduction in travel and emergency care costs, better use of resources, secondary improved monitoring of chronic patients, and finally reduced risk of infection and hospital overcrowding. Other interesting advantage described were: rapidity, simplicity of the teleconsult; utility for follow up; greater empathy between hospital and family and new method of prophylaxis and screening of pathologies.

Lastly, we evaluated the opinion of scientific societies on the possible future effects of the SARS-Cov-2 pandemia in pediatric medical assistance. Most scientific societies believe that they will treat COVID-19 related diseases, primarily deriving from the indirect action of the virus, mostly psychosomatic disorders. In fact, during a severe pandemic like COVID-19, community-based mitigation programs, such as closing of schools, parks, and playgrounds will disrupt children’s usual lifestyle and can potentially promote distress, confusion, anxiety and hostility [19].

In this context, we think that it is necessary to start again with a shared strategy to ensure an efficient management and continuity of care for pediatric patients. The most of scientific societies think that it will be necessary to formulate a new approach for hospitalizations and outpatient visits. But, in which way?

Firstly, through greater use of telemedicine for the monitoring of patients with chronic diseases. Secondary, it is crucial eliminate the fear of COVID-19 infection, through information (implementing awareness, campaigns regarding vaccinations and the use of personal protective equipment) and creating “clean” hospital with clean pathway for infected and non-infected children. Furthermore, it will be important to act on families through educational program on red flags of pathologies and autonomous first basic level management of children diseases (i.e.fever). At last, all scientific societies agree that family pediatricians will have a more decisive role in the care of the child, to ensure adequate continuity of care and an effective filter function, identifying the real need for hospitalization and specialist visits, creating a new model of territorial assistance.

## Conclusion

The COVID-19 pandemia and subsequent assistance events detected during the lockdown have highlighted many aspects that can be improved in pediatric care, pointing out the need for a scientific conference with the aim to remodulate the pediatric health care, developing a program starting from the family and reaching the hospital through a new and efficient model of primary care.

## Supplementary information

**Additional file 1.** Fiarped questionnaire.

## Data Availability

Not applicable.

## Abbreviations

COVID-19: Coronavirus disease 2019
FIARPED: Federation of Associations and Scientific Societies of the Pediatric Area

## References

[1] WHO Director-General's opening remarks at the media briefing on COVID-19 - 11 March 2020. https://www.who.int/dg/speeches/detail/who-director-general-s-opening-remarks-at-the-media-briefing-on-covid-19%2D%2D-11-march-2020.

[2] Liu W, Zhang Q, Chen J, Xiang R, Song H, Shu S (2020). Detection of Covid-19 in children in early January 2020 in Wuhan, China. N Engl J Med.

[3] Xu Y, Li X, Zhu B, Liang H, Fang C, Gong Y (2020). Characteristics of pediatric SARS-CoV-2 infection and potential evidence for persistent fecal viral shedding. Nat Med.

[4] Chen ZM, Fu JF, Shu Q, Chen YH, Hua CZ, Li FB (2020). Diagnosis and treatment recommendations for pediatric respiratory infection caused by the 2019 novel coronavirus. World J Pediatr.

[5] Mengoni A, Rappini V (2007). La domanda non urgente al Pronto Soccorso: un’analisi. Mecosan.

[6] Carret ML, Fassa AC, Domingues MR (2009). Inappropriate use of emergency services: a systematic review of prevalence and associated factors. Cad Saude Publica.

[7] Vedovetto A, Soriani N, Merlo E, Gregori D (2014). The burden of inappropriate emergency department pediatric visits: why Italy needs an urgent reform. Health Serv Res.

[8] Ministero della Salute. Le caratteristiche dell’ospedalizzazione pediatrica in Italia dal neonato all’adolescente: http://www.ministerosalute.it/resources/static/primopiano/206/documento.pdf.

[9] P.R.U.O. – Protocollo per la revisione dell’uso dell’ospedale. Progetto Ministeriale. http://docplayer.it/13771683-P-r-u-o-progettoministeriale.html.

[10] Esmail A, Quayle JA, Roberts C (2000). Assessing the appropriateness of pediatric hospital admissions in the United Kingdom. J Public Health Med.

[11] Bianco A, Pileggi C, Trani F, Angelillo IF (2003). Appropriateness of admissions and days of stay in pediatric wards of Italy. Pediatrics.

[12] Chiaradia G, de Waure C, La Torre G, Paparatti M, Ricciardi W (2008). Appropriateness of admission and hospital stay in pediatric wards: the case of a teaching hospital in Rome. Ann Ig.

[13] Parizzi F, D’Andrea N, Mastroiacovo P (2006). Appropriatezza dei ricoveri in Pediatria. Studio prospettico multicentrico nell’anno 2003. Quaderni acp.

[14] Vincitorio D, Chiaradia G, Waure C, Mastaki Kambale J, Torre G, Di Stanislao F (2010). Appropriateness of admission and days of stay in pediatric hospital in Ancona, Italy. J Public Health Med.

[15] Sheikhtaheri A, Kermani F (2018). Telemedicine in diagnosis, treatment and Management of Diseases in children. Stud Health Technol Inform.

[16] Burke BL, Hall RW (2015). Telemedicine: pediatric applications. Pediatrics.

[17] Graf N, Paulussen M, Huf T, Ganslandt T, Stahl J, Jürgens H (2002). Telemedicine in pediatric oncology. Klin Padiatr.

[18] Lo MD, Gospe SM (2019). Telemedicine and child neurology. J Child Neurol.

[19] Dubey S, Biswas P, Ghosh R, Chatterjee S, Dubey MJ, Chatterjee S (2020). Psychosocial impact of COVID-19. Diabetes Metab Syndr.

