# Supplementary material for: Point of view of the Italians pediatric scientific societies about the pediatric care during the COVID-19 lockdown: what has changed and future prospects for restarting
Source: Ital J Pediatr. 2020 Oct 2;46:142. doi: 10.1186/s13052-020-00907-3 (PMC7531060; doi:10.1186/s13052-020-00907-3)
Supplement: Supplementary file 1 — Additional file 1. Fiarped questionnaire. [file 13052_2020_907_MOESM1_ESM.docx]

**Attachment 1: Fiarped questionnaire**

**FIARPED**

1. How much do you think the hospitalization activity related to the pathologies of interest of your scientific society is currently reduced?

10 20 30 40 50 60 70 80 90 100 %

1. How much do you think the outpatient activity related to the pathologies of interest of your scientific society is currently reduced?

10 20 30 40 50 60 70 80 90 100 %

1. Which is the serious degree of diseases currently admitted?

Low 0 1 2 3 4 5 high

1. How much do you think the new diagnoses reduced since the introduction of  [laws of containment](https://context.reverso.net/traduzione/inglese-italiano/laws+of+containment)  of March?

10 20 30 40 50 60 70 80 90 100 %

1. What are the reasons of the reduction of hospitalizations?
2. The fear of infection
3. The revaluation by families about the clinical conditions which determine the need for hospitalization
4. failure to send by [family pediatrician](https://context.reverso.net/traduzione/inglese-italiano/family+pediatrician)
5. What are the reasons of the reduction of the outpatient activity?
6. The fear of infection
7. The revaluation by families about the clinical conditions which determine the need for hospitalization
8. failure to send by [family pediatrician](https://context.reverso.net/traduzione/inglese-italiano/family+pediatrician)
9. How much do you think that the hospitalization were inappropriate before of  [laws of containment](https://context.reverso.net/traduzione/inglese-italiano/laws+of+containment),  compared to the reduction of hospitalizations that you reported?

10 20 30 40 50 60 70 80 90 100 %

1. How much consultant’s activity of in the inpatient wards decreased in this period?

10 20 30 40 50 60 70 80 90 100 %

1. How much your consultancy activity for other wards decreased in this period?

10 20 30 40 50 60 70 80 90 100 %

1. How much do you think that the outpatient activity was inappropriate before of  [laws of containment](https://context.reverso.net/traduzione/inglese-italiano/laws+of+containment),  compared to the reduction of outpatient visit that you reported?

10 20 30 40 50 60 70 80 90 100 %

1. Do you think that telemedicine can be a valid tool to replace specialist visits?
2. yes
3. no
4. If you answered yes to the previous question, which telemedicine methods did you use or will you use in the future?
5. Which is the role that telemedicine can play for your specialist?
6. only for verbal consultation
7. to be an active part of an objective examination, what elements do you think can be detected
8. to be an active part of monitoring of one or more instrumental parameters
9. to be an active part of monitoring of one or more biochemical parameters
10. How much do you think the telemedicine can replace your outpatient visit?

10 20 30 40 50 60 70 80 90 100 %

1. Who do you think telemedicine can be useful for?
2. All the children
3. Only patients with chronic conditions
4. Advantages of telemedicine
5. How did you maintaine the relationship with your chronic patients in this period?
6. [according to the timetable](https://context.reverso.net/traduzione/inglese-italiano/according+to+the+timetable) of outpatient visit agreed
7. at the demands of your centers
8. to [specific requests from](https://context.reverso.net/traduzione/inglese-italiano/specific+requests+from+employers) patients
9. How did you continue the care of your chronic patients in this period?
10. [according to the timetable](https://context.reverso.net/traduzione/inglese-italiano/according+to+the+timetable) of outpatient visit agreed in the hospital
11. through outpatient visit agreed in non-hospital structure
12. by phone
13. with video call
14. with app
15. other
16. What degree of clinical safety do you think the contact model adopted above could have provided?

Low 0 1 2 3 4 5 high

1. As you think, will change the normal practices for hospitalizations at the end of the containment?
2. everything will be like before
3. It will be necessary to formulate a new approach methodology by specialist services
4. The [family pediatrician](https://context.reverso.net/traduzione/inglese-italiano/family+pediatrician) will have to play a more decisive role regarding hospitalization needs
5. Other
6. As you think, will change the normal practices for outpatient visit at the end of the containment?
7. everything will be like before
8. It will be necessary to formulate a new approach methodology by specialist services
9. The [family pediatrician](https://context.reverso.net/traduzione/inglese-italiano/family+pediatrician) will have to play a more decisive role regarding hospitalization needs
10. Other
11. Do you think that your specialty will have to treat diseases related to coronavirus infection in the post containment period?
12. yes by the direct action of the virus; which?
13. yes by the indirect action of the virus; which?
14. Do you think will be necessary an educational program for families about the relationship between hospital and regional pediatric services for the post containment?
15. yes
16. no

24) What topics do you think need to be addressed?
